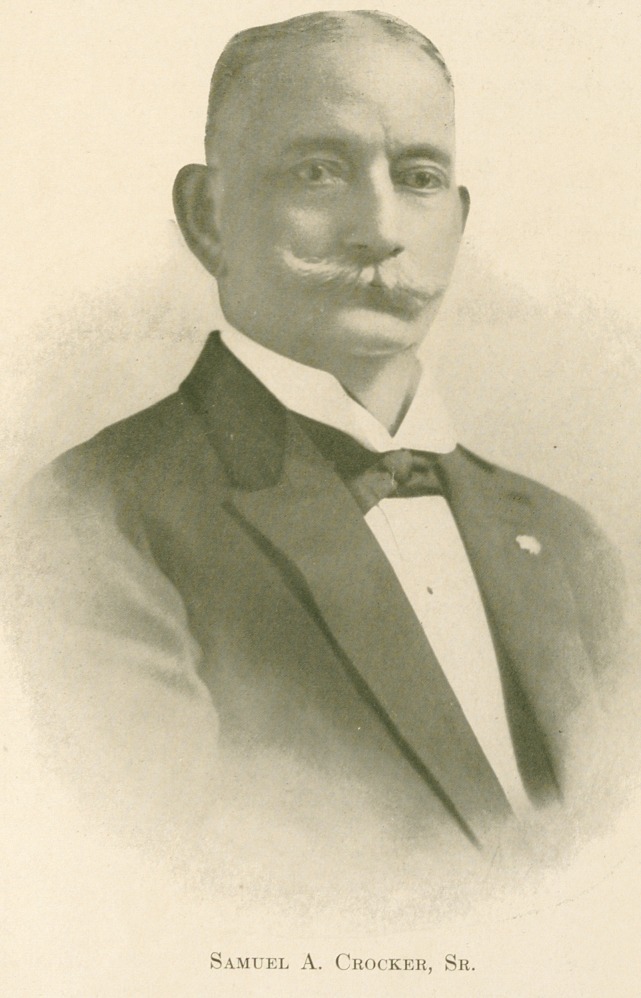# Samuel A. Crocker, Sr.

**Published:** 1913-06-15

**Authors:** 


### OBITUARY.

### Samuel A. Crocker, Sr.

Mr. Samuel A. Crocker, Sr., who was President of The Samuel A. Crocker Company, Publishers of the *Dental Register*, and who built up the dental business of the present corporation from the early days of 1872, was born November 30th, 1844, at Doylestown, Ohio, near Cleveland. Mr. Crocker served three years in the Civil War of 1863, under General Thomas, serving part of the time with the First Ohio Sharpshooters, and part of the time as special mail messenger for General Thomas. This service took him through the battles of Missionary Ridge, Lookout Mountain, Chicamauga, Stone River and elsewhere in the South. But eleven veterans now remain of his company. In 1870 he married Miss Lucy Smith, daughter of Mr. Enoch Noble Smith of Springfield, Massachusetts, and lived in Cleveland, Ohio, and at that time was connected with the firm of Strong, Cobb & Company, wholesale druggists. From there he came to Cincinnati, Ohio, and established the present Dental Business which bears his name. Mr. Crocker was one of the pioneers in the Dental Supply Business, entering the business when it was in its infancy. As a member of the Masons he reached the highest ranks in this order, becoming a Thirty-Second Degree member and a Shriner; he also was a life member of the Elks. Both these organizations have shown him attention and sympathy during his last illness.

Outside of business, to which Mr. Crocker was very devoted, he was a thorough horseman, being a lover of horses, and before the advent of motors, owned some of the best trotters in this vicinity. In this manner he took his pleasure driving to and from his business. His adroit action, upright spirit, and his genial character made friends everywhere, and to-day the name of Crocker is known in every country where dental supplies are sold.

Mr. Crocker died May 21st, 1913, in his 69th year. and is buried in the family lot in Spring Grove Cemetery. He is survived by his widow, Mrs. Sam'l A. Crocker, Sr., and by his two sons, Alfred A. Crocker, and Sam'l A. Crocker, Jr. Both the sons have been with the company since 1897, sixteen years, and remain identified in the management of the business.

SAMUEL A. CROCKER, SR.

## Figures and Tables

**Figure f1:**